# Jaguar interactions with pumas and prey at the northern edge of jaguars’ range

**DOI:** 10.7717/peerj.2886

**Published:** 2017-01-18

**Authors:** Carmina E. Gutiérrez-González, Carlos A. López-González

**Affiliations:** 1 Facultad de Ciencias Naturales, Universidad Autónoma de Querétaro, Querétaro, México; 2 Northern Jaguar Project, Tucson, AZ, USA

**Keywords:** *Panthera onca*, *Puma concolor*, Sonora, México, Two species conditional occupancy model, Species interaction factor, Jaguar, Puma, Activity patterns

## Abstract

We present the first study that evaluates jaguar-puma interactions in the arid lands of northern Mexico, where jaguars have their northernmost breeding population and both predators are persecuted for livestock depredation. We tested whether jaguars are the dominant species in this unique ecosystem, where: (1) pumas outnumber jaguars, (2) pumas are better adapted to arid environments, and (3) jaguars and pumas are of similar size. We analyzed four years of data with two approaches; a two species conditional occupancy model and an activity patterns analysis. We used camera location and prey presence as covariates for jaguar and puma detection and presence probabilities. We also explored overlap in activities of predators and prey. Where both species were detected, peccary presence was positively correlated with both jaguar and puma presence, whereas in areas where jaguars were detected but pumas were not, deer presence explained the probability of jaguar presence. We found that both predators were more likely to co-occur together than to be found independently, and so we rejected the hypothesis that jaguars were the dominant species in our study area. Predators were mainly nocturnal and their activity patterns overlapped by 60%. Jaguar, as compared with puma, overlapped more with deer and calves; puma overlapped with calves more than with other prey, suggesting a preference. We believe exploring predator relationships at different scales may help elucidate mechanisms that regulate their coexistence.

## Introduction

Jaguars (*Panthera onca* Linnaeus, 1758) and pumas (*Puma concolor* Linnaeus, 1771) are the two largest felids in the Americas (Iriarte et al., 1990). Jaguar range overlaps entirely with puma range (Haines, 2006) and their diets also overlap (Nuñez, Miller & Lindzey, 2000; Oliveira, 2002; Scognamillo et al., 2003; Gómez-Ortiz, 2010), especially when prey are abundant (Polisar et al., 2003). Species with overlapping energetic and resource requirements are assumed to have co-evolved mechanisms to minimize competition (Ramesh et al., 2012). Some authors suggest that jaguar and puma coexistence is possible due to dietary segregation (Cascelli de Azevedo, 2008; Foster, Harmsen & Doncaster, 2010). When they overlap, jaguars tend to consume larger prey than pumas, and pumas tend to have a more diverse diet (Iriarte et al., 1990; Polisar et al., 2003; Scognamillo et al., 2003). However, this theory has not been supported in all studies (Nuñez, Miller & Lindzey, 2000; Harmsen et al., 2009).

Studies on feeding ecology of both species have revealed high dietary overlap, but with a higher specialization by jaguars for peccary species (collared peccary, *Pecari tajacu* Linnaeus, 1758 and white-lipped peccary, *Tayassu pecari* Link, 1795) (Oliveira, 2002; Cascelli de Azevedo, 2008) and pumas for deer species (e.g., *Odocoileus* spp.) (Iriarte et al., 1990). Cattle (*Bos taurus* Linnaeus, 1758), especially calves, have also been documented as important prey for both species (Cascelli de Azevedo & Murray, 2007; Rosas-Rosas & Valdez, 2010), leading to conflicts with humans (Cascelli de Azevedo, 2008). Calves are especially vulnerable because they lack natural defensive behaviors, usually roam freely, and represent an easier prey for predators compared with natural prey (Sunquist & Sunquist, 2002; Cascelli de Azevedo & Murray, 2007; Cascelli de Azevedo, 2008; Laundré & Hernández, 2010). López-González & Miller (2002) concluded that jaguars can use both medium and large-sized prey if such prey are available and behaviorally vulnerable; this would include cattle as potential prey.

Temporal segregation has also been suggested as one of the mechanisms that facilitate coexistence (Emmons, 1987; Harmsen et al., 2009). Some authors have found that jaguars are more nocturnal than pumas (Romero-Muñoz et al., 2010; Hernández-Saintmartín et al., 2013), but others have not found differences in their activity patterns (Scognamillo et al., 2003; Harmsen et al., 2009; Paviolo et al., 2009; Foster et al., 2013). This latter theory suggests that predators match the activity of their main prey (Mendes Pontes & Chivers, 2007; Romero-Muñoz et al., 2010); consequently, prey specialization would allow different predators to coexist (Scognamillo et al., 2003).

One hypothesis of competition suggests that larger predators are dominant over smaller-bodied predators. Body size influences the outcomes of interference interactions, with large-bodied carnivores dominating by means of displacing smaller ones from prey abundant habitat patches or prey carcasses (Donadio & Buskirk, 2006). Jaguars living in wet tropical forests are bigger than pumas (Emmons, 1987), and therefore assumed to be the dominant species, especially when jaguars dominate in abundance (Sollmann et al., 2012) and pumas tend to avoid them (Scognamillo et al., 2003). In a dry forest of Bolivia, Romero-Muñoz et al. (2010) evaluated the temporal separation between jaguars and pumas and found that they showed temporal partitioning and that pumas were more abundant than jaguars. They concluded, based on their results, that jaguars did not dominate pumas in three of four study sites probably because of the high densities of pumas in the area and their better adaptation to arid environments.

The interaction patterns for puma and jaguar have been poorly studied in Mexico (Hernández-Saintmartín et al., 2013), but determining patterns that explain their coexistence is important for areas of conservation concern. Northwestern Mexico holds the northernmost reproductive jaguar population reported in the Americas (Brown & López-González, 2001). In this area, the jaguar population has the lowest density reported for the species (Gutiérrez-González et al., 2012) and is subject to extreme environmental conditions (Brown & López-González, 2001) with low survival rates (Gutiérrez-González et al., 2015). Pumas are better adapted to arid environments (Logan & Sweanor, 2001), they outnumber jaguars in this region (Brown & López-González, 2001), and their body size is almost the same as jaguars (Sunquist & Sunquist, 2002). Under the described scenario, we expect pumas to have an advantage over jaguars rather than for jaguars to dominate pumas. Thus, our objective was to evaluate the jaguars’ dominance over pumas in the arid regions of northern Mexico, where we tested for spatial and temporal overlap, and prey activity as explanatory variables for their presence and activity patterns.

## Methods

### Study area

**Figure 1:**
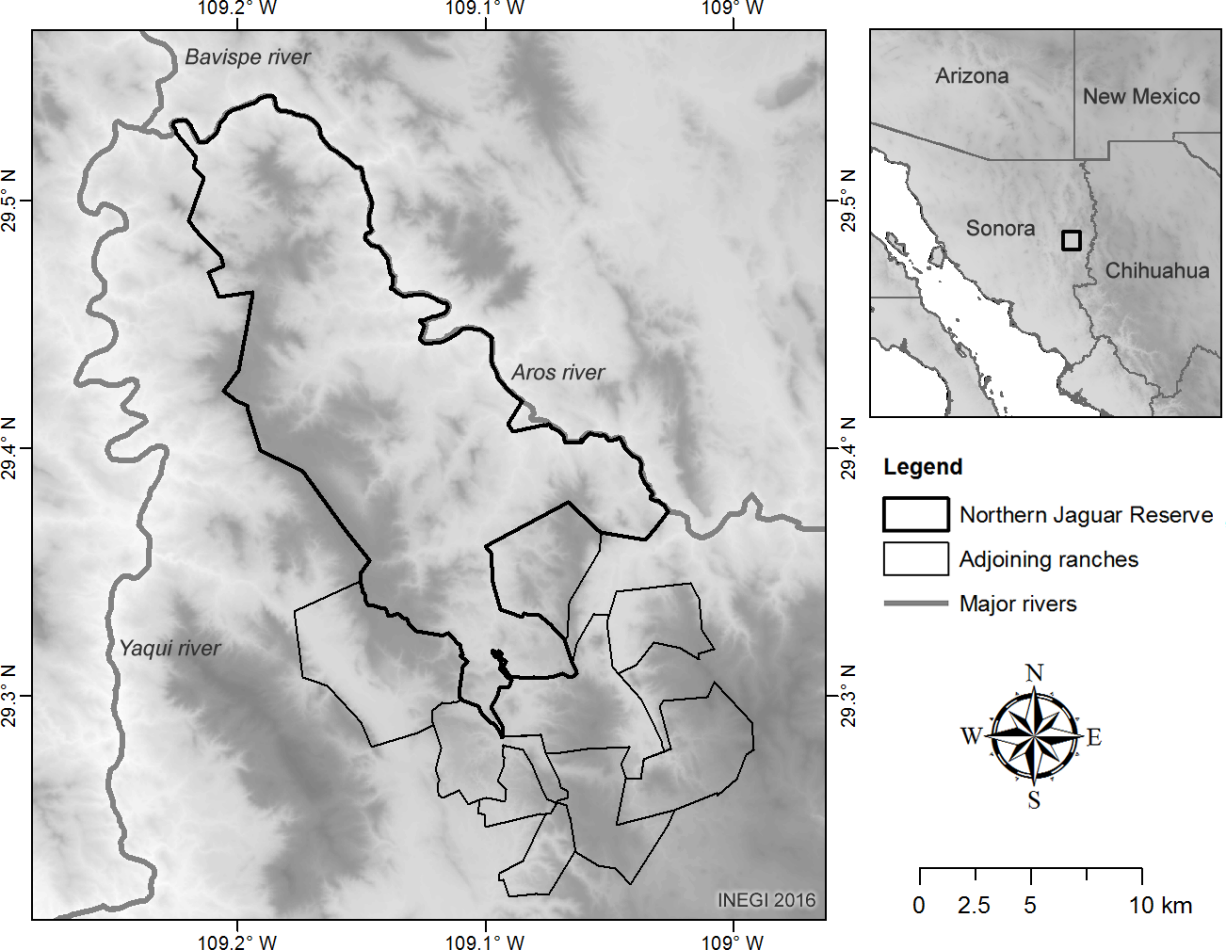
Study area. The study area was composed by the Northern Jaguar Reserve and 10 private cattle ranches. Because of the proximity of the cattle ranches with the private reserve, we considered all properties as a unique study area. The DEM used in this figure can be freely downloaded from www.inegi.gob.mx.

We conducted our study on a privately owned protected area, the Northern Jaguar Reserve, surrounded by privately owned cattle ranches between the coordinates 29°32.4′N–109°14.4′W and 29°12′N–108°58.8′W in northeastern Sonora, Mexico. The area presents altitudes ranging from 370 to 1,600 m and is surrounded by two major rivers (Fig. 1). Annual precipitation ranges between 400 and 800 mm (CONABIO, 2004) and mean temperature is over 18 °C with extreme cold and hot temperatures in winter and summer, −7 °C–46  °C respectively. Vegetation types include desert scrub and thornscrub with tropical affinity (Felger, Johnson & Wilson, 2001). Tropical deciduous forest is present in some canyons and shaded hillsides. Oak woodlands (*Quercus* spp.) are found at elevations >1,000 m and in moist shaded canyons. Native vegetation is mixed with human-induced grassland patches as well as *Dodonaea viscosa* (Felger, Johnson & Wilson, 2001; CONAFOR, 2014).

The private wildlife reserve was created in 2003 with binational collaboration from a Mexican NGO, Naturalia A.C., and an American NGO, the Northern Jaguar Project. In 2007, some cattle ranches also signed a conservation agreement to protect wildlife. Inclusive of the cattle ranches and the private reserve, our study area was approximately 33,000 ha.

### Field work

For the present study we used camera trap data gathered from 2009 to 2012. Camera availability, number, type, and model of camera used in the field changed from year to year (Table 1). Camera traps were separated by ≥1 km and placed in streams, roads, and trails used by wildlife. We changed the location of cameras throughout the study to maximize detections. We set all cameras to have a five-minute delay between capture events and recorded photos 24 h a day. When camera availability allowed, we placed cameras in pairs. No bait or lures were used during the study. We checked cameras monthly.

**Table 1:** Number and model of camera traps in the field from 2009 to 2012. The number and the model of cameras used changed by year due to camera availability. Numbers correspond to one sampling period of one month by year.

Camera model		2009	2010	2011	2012
Camtrakker^®^ 35 mm	Film camera	18	10	–	–
Cuddeback^®^	Digital camera	17	67	85	93
Moultrie^®^	Digital camera	3	–	–	–
Wildview^®^	Digital camera	38	31	12	26

### Data analysis

Wildlife and livestock pictures from January 2009 to September 2012 were archived in an Excel^®^ database for analysis.

#### Two species occupancy model

Jaguar and puma spatial interaction analysis was performed using a *single season conditional two species occupancy model*. This approach requires the designation of one species as dominant. Following assumptions in the literature, we selected jaguar as the dominant species (species A). The two species conditional occupancy model (Richmond, Hines & Beissinger, 2010) includes eight types of parameters that can be separated into (1) occupancy parameters: *ψ*^A^ is the probability of occupancy for species A; *ψ*^BA^ is the probability of occupancy for species B, given species A is present; *ψ*^Ba^ is the probability of occupancy for species B, given species A is absent, and (2) detection parameters: p^A^ is the probability of detection for species A, given species B is absent; p^B^ is the probability of detection for species B, given species A is absent; r^A^ is the probability of detection for species A, given both species are present; r^BA^ is the probability of detection for species B, given both species are present and species A is detected; r^Ba^ is the probability of detection for species B, given both species are present and species A is not detected.

The Species Interaction Factor (SIF) is obtained as a derived parameter. If the SIF = 1, the two species occur independently; if SIF <1, species B is less likely to co-occur with species A than expected under a hypothesis of independence. If SIF >1, species B is more likely to co-occur with species A than expected under a hypothesis of independence (Richmond, Hines & Beissinger, 2010).

In order to meet the demographic closure assumption (Richmond, Hines & Beissinger, 2010), we selected one month from each year to develop the capture histories and considered each day as a sampling session. Other authors have used one sampling month with camera traps for spatial–temporal feline pattern analysis (Carter et al., 2015).

Due to low jaguar detection (Table 2), we pooled sex information and chose the month with the most jaguar records for our analysis. In order to increase our sample size, we considered each camera station as a sampling location; however we recognize that using this approach violates the geographic closure assumption of occupancy models. Therefore, we will refer to presence instead of occupancy in the area (Mackenzie et al., 2006). As occupancy models do not require individual identification, we considered one picture of each species for each surveyed day as a single detection (Sollmann et al., 2012). Each sampling year was modeled as a group. Changes in camera location between years prevented us from using a robust design model (Miller et al., 2012).

**Table 2:** Number of pictures by species by year taken with camera traps. Numbers in parenthesis represent independent events used for activity patterns.

Year	*Panthera onca*	*Puma concolor*	*Odocoileus virginianus*	*Pecari tajacu*	*Bos taurus*^a^
2009	11 (7)	26 (22)	80 (66)	7 (3)	12 (15)
2010	12 (10)	80 (68)	183 (157)	7 (6)	28 (25)
2011	18 (31)	72 (59)	268 (240)	13 (13)	178 (148)
2012	39 (28)	71 (60)	152 (124)	18 (18)	49 (37)

**Notes.**

a Calves only.

We used camera location and prey species as covariates that could explain puma or jaguar detection and presence: Camera location—We used this as a dummy covariate in order to account for possible bias in species detection due to its location on roads (Maffei et al., 2011). Prey species—For each prey species, we used the proportion of the days with pictures by sampling unit by year as a measure of species presence in the site (O’Brien, 2011; Ramesh et al., 2012; Carter et al., 2015). White-tailed deer (*Odocoileus virginianus* Zimmermann, 1780) and collared peccary have been described as the main prey species for pumas and jaguars in areas where they coexist (Oliveira, 2002; Cascelli de Azevedo, 2008). We also included calves as a potential prey for both species (Brown & López-González, 2001; Rosas-Rosas & Valdez, 2010). Felids prefer calves to adult cows (Shaw, 1977; Tortato et al., 2015).

We used an *ad hoc*, stepwise approach for model construction (Richmond, Hines & Beissinger, 2010). We assumed that the detection of one species did not vary in the sampling month, and that our models were time-constant for all detection probabilities.

We tested hypotheses regarding the co-occurrence and detection of puma and jaguar. Specifically, we addressed (1) whether the species co-occurred independently or if there was evidence of competitive exclusion of pumas from sites used by jaguars, (2) if the detection process was independent between species, and (3) if the presence of one species influenced the detection of the second species.

We first evaluated those models that may be related to the detection probabilities by each species (Sollmann et al., 2012). After we had a best adjusted model for detection, we started modeling occupancy parameters, first for jaguar and then for puma. For a full list of models and hypotheses see Table S1. Model selection was based on AICc criteria (Burnham & Anderson, 2002). All models construction was performed using program MARK 8.0 (White & Burnham, 1999).

#### Activity patterns

We compared activity patterns of jaguar, puma and three prey species, using the same dataset for occupancy analysis. To avoid autocorrelation, when it was not possible to identify individuals, we considered one hour between photographs as independent events (Paviolo et al., 2009). We determined the exact time of sunset and sunrise, using the time of the day, the Julian date, and the camera location. We calculated solar time using sunset and sunrise information by year (Perpiñán, 2012).

We used kernel density estimates (Ridout & Linkie, 2009) to generate the activity pattern for each species by year. We classified the activity into three categories by integrating the area under the curve for each period: diurnal (activity predominantly between 1 h after sunrise and 1 h before sunset), nocturnal (activity predominantly between 1 h after sunset and 1 h before sunrise), and crepuscular (activity from 1 h before and after sunrise and sunset) (Foster et al., 2013; Hernández-Saintmartín et al., 2013). This classification corresponded to the probability of observing the animal during that time period (Linkie & Ridout, 2011).

Then, we calculated the coefficient of overlap (Δ1) and its 95% confidence intervals from 10,000 bootstrap samples (Meredith & Ridout, 2014) for each pair of species (jaguar-puma, jaguar-prey, puma-prey) by year. Overlap values range from 0 (no overlap) to 1 (complete overlap). All activity pattern analysis were conducted in program R (R Development Core Team, 2015) using the packages RAtmosphere (Biavati, 2014), solaR (Perpiñán, 2012), and overlap (Meredith & Ridout, 2014).

## Results

We obtained a total of 39,167 pictures from all five species (jaguar, puma, calves, deer, and peccary). With the one month selection criteria, we used 1,324 pictures for the occupancy analysis and 1,137 pictures for the activity patterns analysis (Table 2).

### Two species occupancy model

We generated 32 models (Table 3) and found:

**Table 3:** Table of the five best models for the two species conditional occupancy model. The models represent the jaguar as the dominant species (species A) and the puma as the subordinate (species B).

Model	AICc	Δ AICc	AICc Weights	Model Likelihood	Number of parameters
Ψ^A^(deer) Ψ^BA^(y*peccary) Ψ^Ba^(y*peccary) p^A^ =r^A^(.) p^B^ =r^BA^ =r^Ba^(.)	2368.11	0	0.89	1	8
Ψ^A^(deer) Ψ^BA^(y*peccary) Ψ^Ba^(y*calf) p^A^ =r^A^(.) p^B^ =r^BA^ =r^Ba^(.)	2373.42	5.30	0.06	0.07	9
Ψ^A^(deer) Ψ^BA^(y*peccary) Ψ^Ba^(.) p^A^ =r^A^(.) p^B^ =r^BA^ =r^Ba^(.)	2377.57	9.45	<0.01	<0.01	6
Ψ^A^(deer) Ψ^BA^(y*peccary) Ψ^Ba^(deer) p^A^ =r^A^(.) p^B^ =r^BA^ =r^Ba^(.)	2377.69	9.57	<0.01	<0.01	7
Ψ^A^(deer) Ψ^BA^(y*peccary) Ψ^Ba^(calf) p^A^ =r^A^(.) p^B^ =r^BA^ =r^Ba^(.)	2378.74	10.62	<0.01	<0.01	7

**Notes.**

Ψ Occupancy probability. We refer to the proportion of sites used by the species because we violated assumption related to geographic closure. Ψ^A^ proportion of sites used by species A. Ψ^BA^proportion of sites used by species B when species A is also present. Ψ^Ba^ proportion of sites used by species B when species A is absent. p^A^ detection probability of species A when species B is absent. p^B^ detection probability of species B when species A is absent. r^A^ detection probability when both species are present. r^BA^detection probability when both species are present but only species A is detected. r^Ba^detection probability due both species are present but species A is not detected. (y) corresponds to each year (modeled as group), (peccary, calf and deer) correspond to the proportion of the days each species was detected. (y*) represents the interaction of the year with each covariate. (.) represents no time or covariate effect for the parameter.

#### Detection probabilities

Puma and jaguar detection probabilities were independent of the presence of the other species and were constant across years.

#### Presence probabilities

Puma presence was dependent on jaguar presence. White-tailed deer was important for jaguar presence in all years. Peccary had a different influence on puma presence by year, even if the jaguar was not present. The SIF value showed that the two predator species were more likely to be detected in the same site than separately, except for 2011 when both species were independent (Table 4).

**Table 4:** Probability estimates obtained from the conditional two species occupancy model applied to jaguar and puma in Sonora, Mexico.

Parameter	2009	2010	2011	2012
Ψ^A^	0.28 ± 0.05	0.28 ± 0.05	0.28 ± 0.05	0.28 ± 0.05
Ψ^BA^	1.00 ± 0.0	1.00 ± 0.0	1.00 ± 0.0	1.00 ± 0.0
Ψ^Ba^	0.28 ± 0.04	0.91 ± 0.03	1.00 ± 0.0	0.28 ± 0.04
Ψ^2^	0.28 ± 0.04	0.28 ± 0.04	0.28 ± 0.04	0.28 ± 0.04
p^A^	0.03 ± 0.01	0.03 ± 0.01	0.03 ± 0.01	0.03 ± 0.01
p^B^	0.04 ± 0.003	0.04 ± 0.003	0.04 ± 0.003	0.04 ± 0.003
r^A^	0.03 ± 0.01	0.03 ± 0.01	0.03 ± 0.01	0.03 ± 0.01
r^BA^	0.04 ± 0.003	0.04 ± 0.003	0.04 ± 0.003	0.04 ± 0.003
r^Ba^	0.04 ± 0.003	0.04 ± 0.003	0.04 ± 0.003	0.04 ± 0.003
SIF	3.53 ± 0.59	1.09 ± 0.03	1.00 ± 0.0	3.53 ± 0.59

**Notes.**

Ψ^A^ proportion of sites used by species A. Ψ^BA^ proportion of sites used by species B when species A is also present. Ψ^Ba^ proportion of sites used by species B when species A is ausent. Ψ^2 ^ proportion of sites used by both species at the same time. p^A^ detection probability of species A when species B is absent. p^B^ detection probability of species B when species A is absent. r^A^ detection probability when both species are present. r^BA^ detection probability when both species are present but only species A is detected. r^Ba^ detection probability when both species are present but species A is not detected. SIF, species interaction factor. Species A is the dominant species, jaguar, species B is the subordinate species, puma.

### Activity patterns

**Figure 2:**
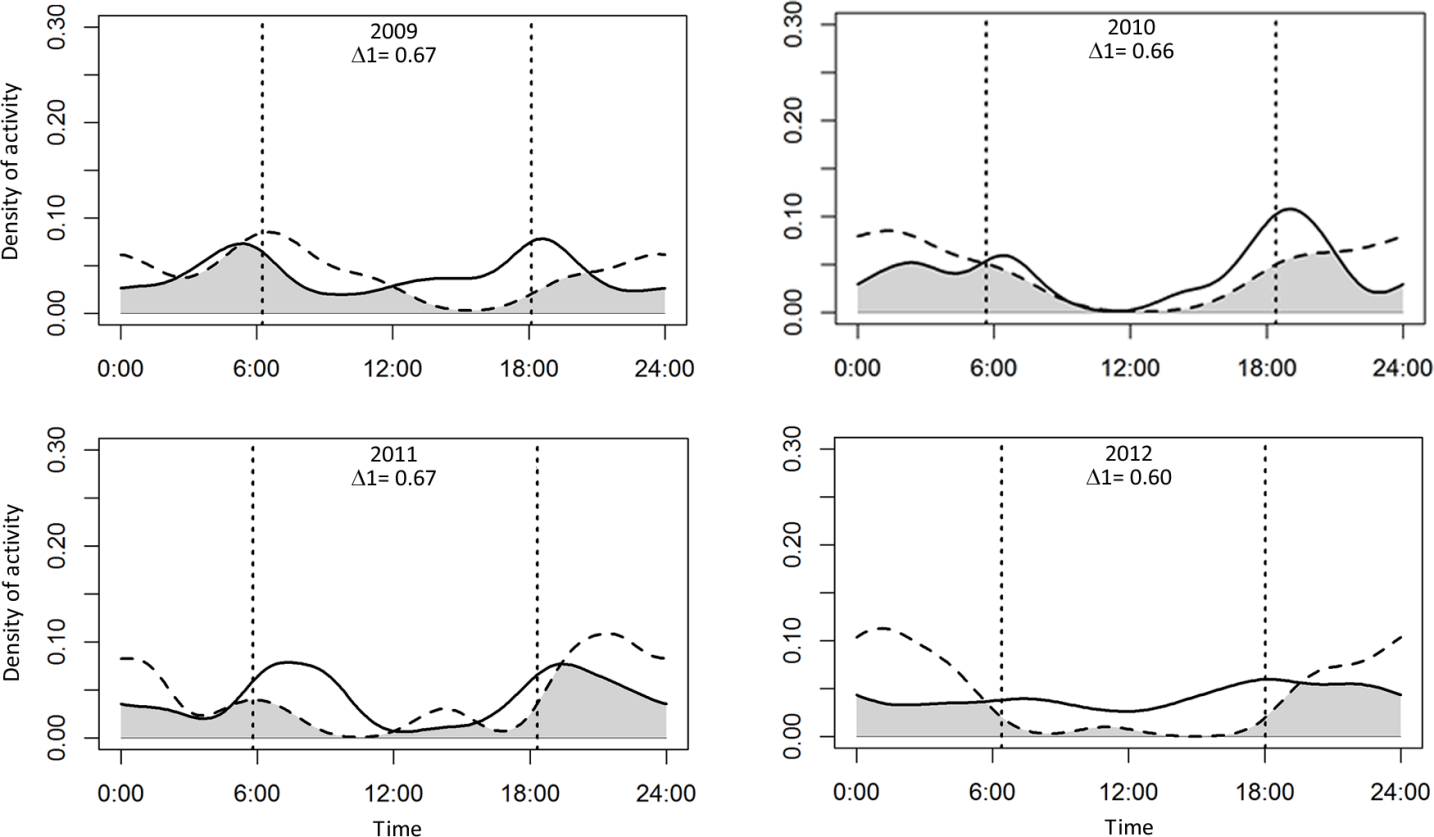
Overlap of daily activity patterns between pumas and jaguars in Sonora, Mexico by year. Overlap is represented by the shaded area. Solid lines represent the activity pattern of pumas and dashed lines represent the activity pattern of jaguars. The vertical dashed lines represent the mean time of sunrise and sunset. Δ1 corresponds to the overlapping coefficient between species activity patterns.

**Figure 3:**
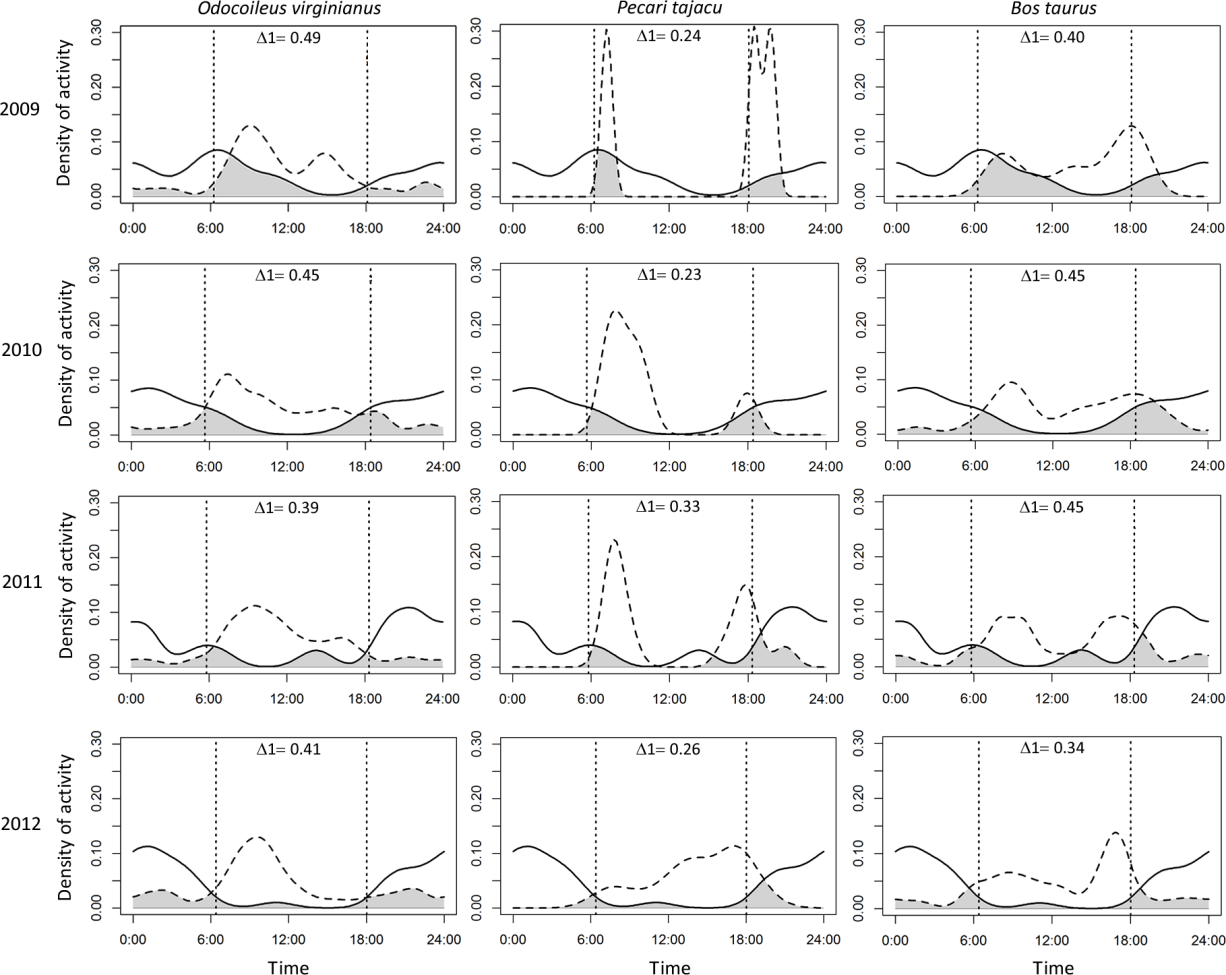
Overlap of daily activity patterns between the jaguar and its main preys in Sonora, Mexico by year. Overlap is represented by the shaded area. Solid lines represent jaguar activity pattern and dashed lines represent the prey activity patterns. Each column corresponds to one prey species and each row corresponds to the sampling year. The vertical dashed lines represent the mean time of sunrise and sunset. Δ1 corresponds to the overlapping coefficient between species activity patterns.

**Figure 4:**
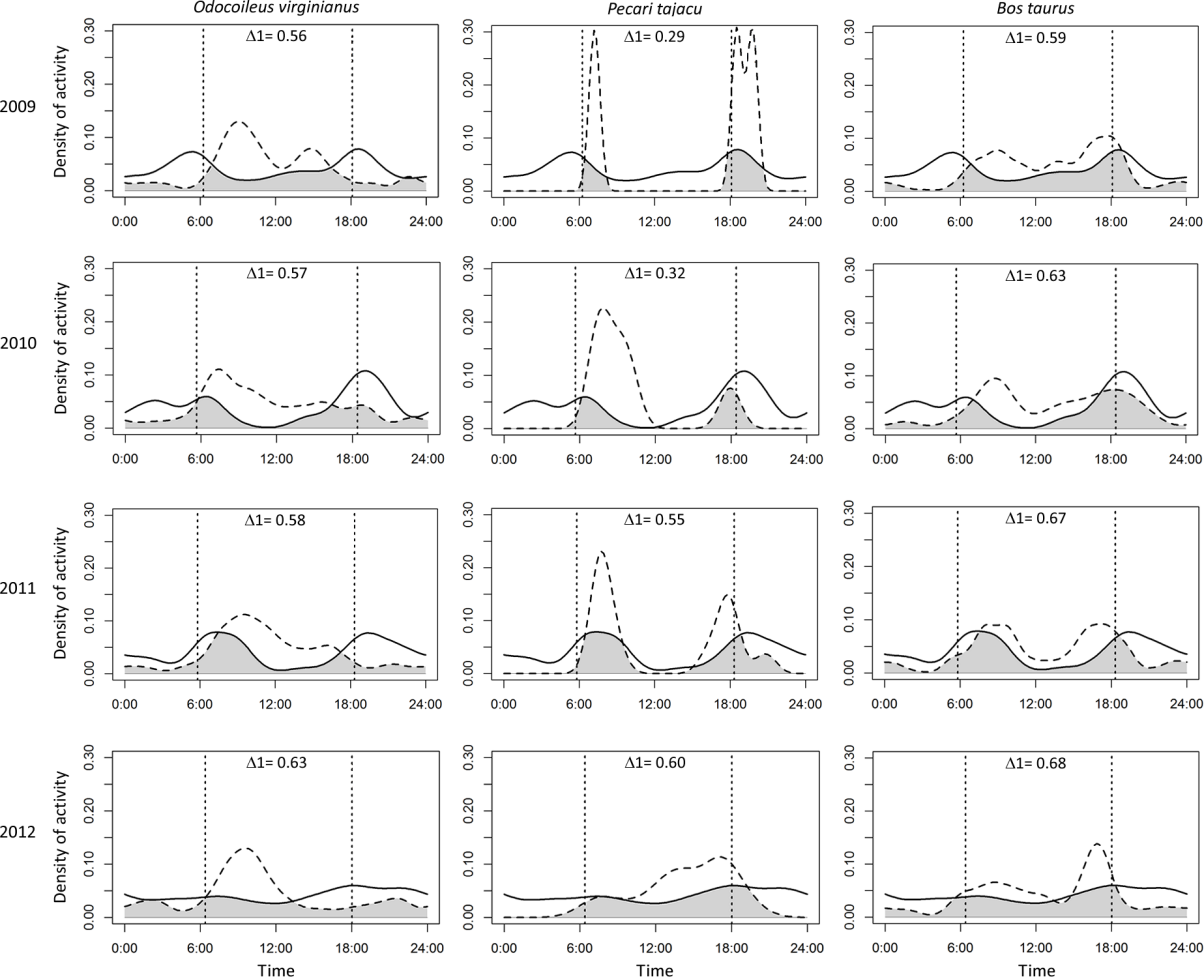
Overlap of daily activity patterns between the puma and its main preys in Sonora, Mexico by year. Overlap is represented by the shaded area. Solid lines represent puma activity pattern and dashed lines represent the prey activity patterns. Each column corresponds to one prey species and each row corresponds to the sampling year. The vertical dashed lines represent the mean time of sunrise and sunset. Δ1 corresponds to the overlapping coefficient between species activity patterns.

Puma and jaguar showed nocturnal activity patterns (Fig. 2), while prey showed mainly diurnal activity, except for 2009 when peccary was more crepuscular than diurnal. Overlapping coefficients for jaguar and puma were on average 0.60 for all years (Fig. 2). Jaguar activity overlapped more with deer activity (mean Δ1 = 0.43) than with peccary (mean Δ1 = 0.26) or calf (mean Δ1 = 0.41) activities in all years (Fig. 3). Puma activity overlapped more with calf activity (mean Δ1 = 0.64) than with deer (mean Δ1 = 0.58) or peccary activity (mean Δ1 = 0.44) (Fig. 4).

## Discussion

We concluded that jaguars were not dominant over pumas in our study area, in contrast to other areas where the species exhibit clear temporal, spatial, or behavioral differences (Novack et al., 2005; Harmsen et al., 2009; Romero-Muñoz et al., 2010). When competitors from different species have similar body sizes and similar diet, encounters and physical confrontations tend to be avoided since an attack carries high risks for both species, even when potential benefits are large (Donadio & Buskirk, 2006; Ramesh et al., 2012; Vanak et al., 2013). Similar-sized competitor species that hunt at the same time of the day can coexist if they hunt in different areas (Ruth & Murphy, 2010). If pumas and jaguars are partitioning the space throughout the day, encounter probabilities decrease and may allow coexistence (Ruth & Murphy, 2010). Large prey species abundance is also an important habitat component that favors the coexistence of large carnivores (Odden, Wegge & Fredriksen, 2010; Mitchell & Hebblewhite, 2012; Carter et al., 2015), and may have supported coexistence in our study area.

Similar to the results of Romero-Muñoz et al. (2010) in a dry tropical forest in Bolivia, we found evidence that pumas were likely to co-occur with jaguars more than expected by chance or that both species were behaving independently (SIF values, Table 4). Our spatial analysis provide stronger evidence that pumas are not avoiding jaguars in dry areas. Further, both pumas and jaguars were active throughout the day (Fig. 2). Similar patterns have also been documented between jaguars and pumas in tropical wet areas of Central America (Davis, Kelly & Stauffer, 2011), as well as between cheetahs (*Acinonyx jubatus* Schreber, 1775) and leopards (*Panthera pardus* Linnaeus, 1758, Vanak et al., 2013), and between tigers (*Panthera tigris* Linnaeus, 1758) and leopards (Carter et al., 2015). Vanak et al. (2013) suggested that interactions between large carnivores were better explained by habitat type and seasonality than the presence of competitive carnivore species.

Although we expected that jaguar activity would be closely related to peccary activity and pumas to be more related to deer activity (Mendes Pontes & Chivers, 2007; Romero-Muñoz et al., 2010), the high overlap of both predators with deer and calf activity is possibly related to a higher abundance of these species in the area (Table 2) (Laundré & Hernández, 2010; Ruth & Murphy, 2010; Hayward et al., 2016). Further, the social behavior of peccaries and their defensive herding strategy may make deer and calves easier prey for both felines (Eisenberg & McKay, 1974; Sunquist & Sunquist, 2002; Scognamillo et al., 2003; Cascelli de Azevedo, 2008). Jaguars can adapt their diet according to prey availability (Cascelli de Azevedo, 2008), and up to 111 species are known to constitute its food habits (Hayward et al., 2016). López-González & Miller (2002) proposed that jaguars consume larger prey size when they are farther north of the Equator. However, a recent analysis proposed that jaguars select prey based on their abundance and herd size rather than their body size (Hayward et al., 2016). When one prey species is more abundant than the other, overlap between predators increases because of the switch in the prey selection of one predator (Ruth & Murphy, 2010; Hayward et al., 2016). But if there is enough prey diversity, the subordinate competitor has no need to avoid the dominant species (Carter et al., 2015).

Pumas have a greater flexibility in prey selection in comparison with jaguars (Iriarte et al., 1990; Polisar et al., 2003; Scognamillo et al., 2003). Due to their flexible diet and better adaptation to arid environments, pumas may exploit a wider array of resources than jaguars, supporting coexistence (Ruth & Murphy, 2010). Predators can also develop different hunting strategies for the same prey species (e.g., selecting individuals of different size) (Murphy & Ruth, 2010; Ruth & Murphy, 2010). Due to the domestication of cattle, jaguars can depredate larger-sized individuals in comparison to their natural prey (Hayward et al., 2016), and pumas tend to select young adults or juveniles (Murphy & Ruth, 2010). Calves have a higher predation risk, especially in places where cattle are allowed to roam loose, and become easier prey for felines than deer (Cascelli de Azevedo & Murray, 2007). Even though puma activity patterns overlapped more with calves (mean Δ1 = 0.64) than did jaguars, we found an overlap of 0.40 on average for jaguars and calves, almost the same overlap that jaguars presented with deer (mean Δ1 = 0.43). Jaguars could be selecting calves and deer in the same proportion if cattle are available and vulnerable in the area (López-González & Miller, 2002; Tortato et al., 2015).

In this study area, as in most ranching areas of Latin America, cattle move freely and unsupervised within the property boundaries without an established breeding season, making calves available throughout the year and more vulnerable to depredation (Laundré & Hernández, 2010; Tortato et al., 2015). Based on the theory that felines tend to follow the activity and movement patterns of their prey (Mendes Pontes & Chivers, 2007; Carrillo, Fuller & Saenz, 2009), the high overlap of puma and jaguar with calves suggest that cattle could be an important element in their diet (Rabinowitz & Nottingham Jr, 1986).

Understanding the mechanisms that regulate the coexistence of pumas and jaguars is especially important in areas where jaguar densities are low because human-feline conflicts often result in retaliatory acts on jaguars, even when pumas were responsible for depredation events (Brown & López-González, 2001). In our study area, jaguars are blamed for predation events and, based on our results, these events could be more related to puma attacks than jaguars.

The two species occupancy model provided us more detailed information about jaguar and puma interactions and their association with prey than activity patterns. Although we found spatial and temporal evidence that pumas are not subordinate to jaguars, and that prey presence and activity patterns played an important role in explaining the presence of both species, we lacked data on actual prey densities or carnivore diet. Based on their detections (Table 2), we considered that prey relative abundance was high and that this factor likely contributed to feline coexistence in our study area (Polisar et al., 2003). An important next step is to study diet overlap in these two top carnivores. For now, however, strategies in the area should be implemented individually for both species taking into consideration our results about prey and predator associations.

##  Supplemental Information

10.7717/peerj.2886/supp-1 Table S1 List of the hypothesis tested in the analysis

10.7717/peerj.2886/supp-2 Data S1 Activity data Information of activity hour and date for each of the five species studied.

10.7717/peerj.2886/supp-3 Data S2 Two species conditional occupancy capture history Sheet 1 includes jaguar-puma detection history used for the analysis. The sheed includes covariate information (deer, cattle, peccary and road).

## Additional Information and Declarations

### Data Availability

The following information was supplied regarding data availability: The raw data has been supplied as a Table S1.

## References

[ref-1] Biavati G (2014). https://cran.r-project.org/web/packages/RAtmosphere/index.html.

[ref-2] Brown DE, López-González CA (2001). Borderland jaguars: tigres de la frontera.

[ref-3] Burnham KP, Anderson DR (2002). Model selection and multimodel inference—a practical information-theoretic approach.

[ref-4] Carrillo E, Fuller TK, Saenz JC (2009). Jaguar (*Panthera onca*) hunting activity: effects of prey distribution and availability. Journal of Tropical Ecology.

[ref-5] Carter N, Jasny M, Gurung B, Liu J (2015). Impacts of people and tigers on leopard spatiotemporal activity patterns in a global biodiversity hotspot. Global Ecology and Conservation.

[ref-6] Cascelli de Azevedo FC (2008). Food habits and livestock depredation of sympatric jaguars and pumas in the Iguaçu National Park Area, South Brazil. Biotropica.

[ref-7] Cascelli de Azevedo FC, Murray DL (2007). Evaluation of potential factors predisposing livestock to predation by jaguars. Journal of Wildlife Management.

[ref-8] CONABIO, Comisión Nacional para el Conocimiento y Uso de la Biodiversidad Conabio (2004). Climas de México.

[ref-9] CONAFOR, Comisión Nacional Forestal (2014). Inventario Nacional Forestal y de Suelos.

[ref-10] Davis ML, Kelly MJ, Stauffer DF (2011). Carnivore co-existence and habitat use in the Mountain Pine Ridge Forest Reserve, Belize. Animal Conservation.

[ref-11] Donadio E, Buskirk SW (2006). Diet, morphology, and interspecific killing in carnivora. The American Naturalist.

[ref-12] Eisenberg JF, McKay GM, Geist V, Walther F (1974). Comparison of ungulate adaptations in the new world and old world tropical forests with special reference to Ceylon and rainforest of Central America. The behaviour of ungulates and its relation to management.

[ref-13] Emmons LH (1987). Comparative feeding ecology of felids in a Neotropical rainforest. Behavioral Ecology Sociobiology.

[ref-14] Felger R, Johnson M, Wilson M (2001). The trees of Sonora, Mexico.

[ref-15] Foster RJ, Harmsen BJ, Doncaster CP (2010). Habitat use by sympatric jaguars and pumas across a gradient of human disturbance in Belize. Biotropica.

[ref-16] Foster VC, Sarmento P, Torres N, Jácomo AT, Negroes N, Fonseca C, Silveira L (2013). Jaguar and puma activity patterns and predator–prey interactions in four Brazilian biomes. Biotropica.

[ref-17] Gómez-Ortiz Y (2010). Nicho trófico de jaguar y puma en la Reserva Natural Sierra Nanchititla, México. MSc Thesis.

[ref-18] Gutiérrez-González CE, Gómez-Ramírez MÁ, López-González CA (2012). Estimation of the density of the near threatened jaguar *Panthera onca* in Sonora, Mexico, using camera trapping and an open population model. Oryx.

[ref-19] Gutiérrez-González CE, Gómez-Ramírez MA, López-González CA, Doherty Jr PF (2015). Are private reserves effective for jaguar conservation?. PLOS ONE.

[ref-20] Haines A (2006). Is there competition between sympatric jaguar *Panthera onca* and puma *Puma concolor*?. Acta Zoologica Sinica.

[ref-21] Harmsen BJ, Foster RJ, Silver SC, Ostro LET, Doncaster CP (2009). Spatial and temporal interactions of sympatric jaguars (*Panthera onca*) and pumas (*Puma concolor*) in a Neotropical forest. Journal of Mammalogy.

[ref-22] Hayward MW, Kamler JF, Montgomery RA, Newlove A, Rostro-García S, Sales LP, Van Valkenburgh B (2016). Prey preferences of the jaguar *Panthera onca* reflect the post-Pleistocene demise of large prey. Frontiers in Ecology and Evolution.

[ref-23] Hernández-Saintmartín AD, Rosas-Rosas OC, Palacio-Núñez J, Tarango-Arámbula LA, Clemente-Sánchez F, Hoogesteijn AL (2013). Activity patterns of jaguar, puma and their potential prey in San Luis Potosi, Mexico. Acta Zoologica Mexicana.

[ref-24] Iriarte JA, Franklin WL, Johnson W, Redford KH (1990). Biogeographic variation of food habits and body size of the America puma. Oecologia.

[ref-25] Laundré JW, Hernández L, Hornocker M, Negri S (2010). What we know about pumas in Latin America. Cougar ecology & conservation.

[ref-26] Linkie M, Ridout MS (2011). Assessing tiger-prey interactions in Sumatran rainforest. Journal of Zoology.

[ref-27] Logan KA, Sweanor LL (2001). Desert puma.

[ref-28] López-González CA, Miller BJ (2002). Do jaguars (P*anthera onca*) depend on large prey?. Western North American Naturalist.

[ref-29] Mackenzie DI, Nichols JD, Royle JA, Pollock KH, Bailey LL, Hines JE (2006). Occupancy estimation and modeling.

[ref-30] Maffei L, Polisar J, Garcia R, Moreira J, Noss AJ (2011). Perspectives from ten years of jaguar (*Panthera onca*) camera trapping in Mesoamerica. Mesoamericana.

[ref-31] Mendes Pontes AR, Chivers DJ (2007). Peccary movements as determinants of the movements of large cats in Brazilian Amazonia. Journal of Zoology.

[ref-32] Meredith M, Ridout M (2014).

[ref-33] Miller DAW, Brehme CS, Hines JE, Nichols JD, Fisher RN (2012). Joint estimation of habitat dynamics and species interactions: disturbance reduces co-occurrence of non-native predators with an endangered toad. Journal of Animal Ecology.

[ref-34] Mitchell MS, Hebblewhite M, Boitani L, Powell RA (2012). Carnivore habitat ecology: integrating theory and application. Carnivore ecology and conservation.

[ref-35] Murphy K, Ruth TK, Hornocker M, Negri S (2010). Diet and prey selection of a perfect predator. Cougar ecology & conservation.

[ref-36] Novack AJ, Main MB, Sunquist ME, Labisky RF (2005). Foraging ecology of jaguar (*Panthera onca*) and puma (*Puma concolor*) in hunted and non-hunted sites within the Maya Biosphere Reserve, Guatemala. Journal of Zoology.

[ref-37] Nuñez R, Miller B, Lindzey F (2000). Food habits of jaguars and pumas in Jalisco, Mexico. Journal of Zoology.

[ref-38] O’Brien TG, O’Connell AF, Nichols JD, Karanth KU (2011). Abundance, density and relative abundance: a conceptual framework. Camera traps in animal ecology.

[ref-39] Odden M, Wegge P, Fredriksen T (2010). Do tigers displace leopards? If so, why?. Ecological Research.

[ref-40] Oliveira TG, Medellín RA, Equihua C, Chetkiewicz CL, Crawshaw Jr PG, Rabinowitz A, Redford KH, Robinson JG, Sanderson EW, Taber A (2002). Ecología comparativa de la alimentación del jaguar y del puma en el geotrópico. El jaguar en el nuevo milenio.

[ref-41] Paviolo A, Di Blanco YE, De Angelo CD, Di Bitetti MS (2009). Protection affects the abundance and activity patterns of pumas in the Atlantic Forest. Journal of Mammalogy.

[ref-42] Perpiñán O (2012). solaR: solar radiation and photovoltaic systems with R. Journal of Statistical Software.

[ref-43] Polisar J, Maxit I, Scognamillo D, Farrell L, Sunquist M, Eisenberg JF (2003). Jaguars, pumas, their prey base, and cattle ranching: ecological interpretations of a management problem. Biological Conservation.

[ref-44] R Development Core Team (2015). R: a language and environment for statistical computing.

[ref-45] Rabinowitz AR, Nottingham Jr BG (1986). Ecology and behaviour of the jaguar (*Panthera onca*) in Belize, Central America. Journal of Zoology.

[ref-46] Ramesh T, Kalle R, Sankar K, Qureshi Q (2012). Spatio-temporal partitioning among large carnivores in relation to major prey species in Western Ghats. Journal of Zoology.

[ref-47] Richmond OMW, Hines JE, Beissinger SR (2010). Two-species occupancy models: a new parameterization applied to co-occurrence of secretive rails. Ecological Applications.

[ref-48] Ridout MS, Linkie M (2009). Estimating overlap of daily activity patterns from camera trap data. Journal of Agricultural, Biological, and Environmental Statistics.

[ref-49] Romero-Muñoz A, Maffei L, Cuéllar E, Noss AJ (2010). Temporal separation between jaguar and puma in the dry forests of southern Bolivia. Journal of Tropical Ecology.

[ref-50] Rosas-Rosas OC, Valdez R (2010). The role of landowners in jaguar conservation in Sonora, Mexico. Conservation Biology.

[ref-51] Ruth TK, Murphy K, Hornocker M, Negri S (2010). Competition with other carnivores for prey. Cougar ecology & conservation.

[ref-52] Scognamillo D, Maxit IE, Sunquist M, Polisar J (2003). Coexistence of jaguar (*Panthera onca*) and puma (*Puma concolor*) in a mosaic landscape in the Venezuelan Llanos. Journal of Zoology.

[ref-53] Shaw H, Phillips RC, Jonkel C (1977). Impact of mountain lion on mule deer and cattle in northwestern Arizona. Proceedings of the 1975 Predator Symposium.

[ref-54] Sollmann R, Malzoni Furtado M, Hofera H, Jácomo ATA, Mundim Torres N, Silveira L (2012). Using occupancy models to investigate space partitioning between two sympatric large predators, the jaguar and puma in central Brazil. Mammalian Biology.

[ref-55] Sunquist M, Sunquist F (2002). Wild cats of the world.

[ref-56] Tortato FR, Layme VMG, Crawshaw Jr PG, Izzo TJ (2015). The impact of herd composition and foraging area on livestock predation by big cats in the Pantanal of Brazil. Animal Conservation.

[ref-57] Vanak AT, Fortin D, Thaker M, Ogden M, Owen C, Greatwood S, Slotow R (2013). Moving to stay in place: behavioral mechanisms for coexistence of African large carnivores. Ecology.

[ref-58] White GC, Burnham KP (1999). Program MARK: survival estimation from populations of marked animals. Bird Study.

