# Supplementary material for: Jaguar interactions with pumas and prey at the northern edge of jaguars’ range
Source: PeerJ. 2017 Jan 18;5:e2886. doi: 10.7717/peerj.2886 (PMC5248577; doi:10.7717/peerj.2886)
Supplement: Table S1 [file peerj-05-2886-s001.doc]

**Appendix 1. List of the hypothesis tested in the analysis.** We selected the jaguar as the dominant species (A). ψ^A^ is the probability of occupancy for species A. ψ^BA^ is the probability of occupancy for species B, given species A is present. ψ^Ba^ is the probability of occupancy for species B, given species A is absent. p^A^ is the probability of detection for species A, given species B is absent. p^B^ is the probability of detection for species B, given species A is absent. r^A^ is the probability of detection for species A, given both species are present. r^BA^ is the probability of detection for species B, given both species are present and species A is detected. r^Ba^ is the probability of detection for species B, given both species are present and species A is not detected

| **Detection parameters** | |
| --- | --- |
| p^A^  p^B^  r^A^  r^BA^  r^Ba^ | Detection of A depends on the presence of B. Detection of B depends on the presence and detection of A. |
| p^A^=r^A^ | Detection of A does not depend on the presence of B. Detection of B depends on the presence and detection of A. |
| r^BA^=r^Ba^ | Detection of A depends on the presence of B. Detecion of B depends on the presence but not on the detection of A. |
| p^A^=r^A^  r^BA^=r^Ba^ | Detection of A does not depend on the presence of B. Detection of B depends on the presence but not on the detection of A. |
| p^A^=r^A^  p^B^=r^BA^=r^Ba^ | Detection of A and B are independent. |
| p^B^=r^BA^=r^Ba^ | Detection of B does not depend on the presence or detection of A. |
| **Occupancy (presence) parameters** | |
| Ψ^A^  Ψ^BA^  Ψ^Ba^ | Occupancy of B depends on the occupancy of A. |
| Ψ^BA^ = Ψ^Ba^ | Occupancy of B does not depend on the occupancy of A. |
| **Covariates** | |
| (.) | Time and year constant. All years with the same probability. |
| (y) | Time constant but different by year. |
| (road) | Camera location effect. Only applied to detection parameters. |
| (deer)  (calves)  (peccary) | Prey effect. Prey are represented as the porportion of days that each prey was detected in the sampling period. Only applied to presence parameters. |
